# Autoimmunity-related *LINC01934* and *AP002954.4* lncRNA polymorphisms may be effective in pediatric celiac disease: a case-control study

**DOI:** 10.1590/1806-9282.20231490

**Published:** 2024-05-03

**Authors:** Seda Orenay-Boyacioglu, Guzide Dogan, Metin Caliskan, Esen Gul Uzuner

**Affiliations:** 1 Aydın Adnan Menderes University, Faculty of Medicine, Department of Medical Genetics, – Aydın, Turkey.; 2 Haseki Education Research Hospital, Department of Pediatric Gastroenterology – İstanbul, Turkey.; 3 Biruni University, Faculty of Medicine, Department of Pediatric Gastroenterology – İstanbul, Turkey.; 4 Uşak University, Faculty of Medicine, Department of Medical Biology – Uşak, Turkey.; 5 Haseki Education Research Hospital, Department of Pathology – İstanbul, Turkey.

**Keywords:** Celiac disease, lncRNA, Polymorphism, Autoimmunity, Epigenetics

## Abstract

**OBJECTIVE:**

Various studies have reported that certain long non-coding RNA levels are unusually low in the intestines of celiac disease patients, suggesting that this may be associated with the inflammation observed in celiac disease. Despite these studies, the research aimed at uncovering the potential role of long non-coding RNAs in the pathogenesis of autoimmune diseases like celiac disease remains insufficient. Therefore, in this study, we plan to assess long non-coding RNA polymorphisms associated with autoimmunity in children diagnosed with celiac disease according to the European Society for Paediatric Gastroenterology Hepatology and Nutrition criteria.

**METHODS:**

DNA was isolated from paraffin tissue samples of 88 pediatric celiac disease patients and 74 healthy pediatric individuals. Single-nucleotide polymorphism genotyping of five long non-coding RNA polymorphisms associated with autoimmunity (*LINC01934-rs1018326*, *IL18RAP-rs917997*, *AP002954.4-rs10892258*, *UQCRC2P1-rs6441961*, and *HCG14 rs3135316*) was conducted using the TaqMan single-nucleotide polymorphism genotyping assays with the LightCycler 480.

**RESULTS:**

In our study, the genotypic and allelic frequency distribution of *LINC01934-rs1018326* and *AP002954.4-rs10892258* polymorphisms was found to be statistically significant in the comparison between the two groups (p<0.05). According to the multiple genetic model analyses, the *LINC01934-rs1018326* polymorphism was observed to confer a 1.14-fold risk in the recessive model and a 1.2-fold risk in the additive model for pediatric celiac disease. Similarly, the *AP002954.4-rs10892258* polymorphism was found to pose a 1.40-fold risk in the dominant model and a 1.7-fold risk in the additive model.

**CONCLUSION:**

Our study results draw attention to the *LINC01934-rs1018326* and *AP002954.4-rs10892258* polymorphisms in celiac disease and suggest that these polymorphisms may be associated with inflammation in autoimmune diseases like celiac disease.

## INTRODUCTION

Celiac disease (CeD) is a food-related small intestine disorder that is also named gluten-sensitive enteropathy and is observed in approximately 1–2% of people. Active CeD is characterized by villous atrophy, crypt hyperplasia, and lymphocytic infiltration in the intestinal epithelium. While it is known that gluten proteins serve as environmental triggers in CeD, the genetic risk factors have not yet been fully defined. It is known that the human leukocyte antigen (HLA) genes are responsible for approximately 40% of the genetic risk for developing CeD, and the majority of CeD patients carry HLA-DQ2 or HLA-DQ8 risk alleles^1–4^. However, both genetic and epigenetic variants within and outside the HLA region are also associated with the risk of developing CeD^3,4^. In this regard, the results of genome-wide association studies (GWAS) have shown that over 85% of the single nucleotide polymorphisms (SNPs) associated with diseases are found in the non-coding parts of the genome. It has been shown that the SNPs found in these regions can regulate the expression of many genes^4^. Therefore, illuminating the disease-associated functional effects of non-coding variants will assist in clarifying the role of immune-related SNPs in disease susceptibility^3^.

Non-coding RNAs over 200 bases are classified as long non-coding RNAs (lncRNAs) and do not encode proteins^5–7^. The lncRNAs can influence processes related to the passage of gluten peptides through the intestinal barrier and the activation of both innate and adaptive immune responses in the pathogenesis of CeD^3^. Many studies have identified lncRNAs associated with CeD. The *AC104820.2* lncRNA was reported as upregulated in the intestinal mucosa of active CeD patients^8^. Another report identified lnc13 as being associated with susceptibility to CeD and demonstrated its functional role^9^. A recent study has shown that gliadin induces the expression of two lncRNAs (*TUG1* and *NEAT1*) in biopsies taken from CeD patients on a gluten-free diet^10^. Plaza-Izurieta et al. and Trynka et al. found an association of *LINC01934-rs1018326* with CeD risk in their studies^8,11^. Additionally, a meta-analysis conducted in 2015 provides strong predictions that *IL18RAP-rs917997* and *UQCR2P1-rs6441961* may be potential risk factors for CeD in European populations^12^. In light of the research conducted in this field, some lncRNAs have been associated with CeD, but there is still a lack of sufficient experimental evidence regarding their effects on the development of this disease. Therefore, in this study, lncRNA polymorphisms related to autoimmunity were investigated in pediatric CeD (pCeD).

## METHODS

### Cases and ethics

The study included 88 pCeD patients who presented to the Pediatric Gastroenterology Clinic of the Department of Pediatrics at Haseki Education Research Hospital in Istanbul. These patients were suspected of having CeD based on the European Society for Paediatric Gastroenterology Hepatology and Nutrition (ESPGHAN) criteria, and their duodenal biopsy samples were histopathologically evaluated as Marsh stage 3^13^. Additionally, 84 healthy children who underwent upper gastrointestinal endoscopy for various reasons and had normal duodenal biopsy results were included in the study as the control group.

The study was conducted upon approval from the Institutional Ethics Board (#2023/254). Informed consent of all participants was obtained before the study.

### SNP selection

In the study, the lncRNA polymorphism associated with five autoimmune diseases to be investigated (*LINC01934-rs1018326*, *lL18RAP-rs917997*, *AP002954.4-rs10892258*, *UQCRC2P1-rs6441961*, and *HCG14-rs3135316*) was determined through a literature review.

### Genomic DNA isolation, concentration, and purity

In our research, genomic DNA isolation from biopsy samples embedded in paraffin blocks was carried out using a commercially available DNA FFPE isolation kit (GeneRead^TM^ FFPE kit, Qiagen, Hilden, Germany). The quality and concentration of the isolated DNA samples were assessed with a spectrophotometer (NanoDrop 1000 V3.7, Thermo Scientific, USA).

### Genotyping

Five SNPs in five different lncRNAs were examined in isolated DNA samples from biopsy specimens. The screening of the lncRNA SNPs was conducted using quantitative real-time polymerase chain reaction (RT-qPCR) with a TaqMan SNP Genotyping Assays (Thermo Fisher Scientific, Waltham, MA). A volume of 10 μL qPCR reaction mixture was prepared, consisting of 0.5 μL TaqMan SNP Genotyping Assay, 5 μL LightCycler 480 Probes Master (Roche Diagnostics KK), 2.5 μL RNase-free water, and 2 μL DNA (50 ng/μL). The qPCR procedure was carried out on a LightCycler480 system (Roche, Germany) with the following conditions: 95°C for 10 min, followed by 45 cycles of 95°C for 15 s, and 60°C for 1 min. Data analysis was conducted using the LightCycler 480 software in the Tm calling mode or with melting curve genotyping.

### Statistical analyses

The chi-squared test was applied for the comparison of categorical variables between the two groups. Student's t-test was employed to compare continuous independent variables. The Hardy-Weinberg equilibrium (HWE) was assessed by comparing the genotype distribution of the subjects with those of the controls, utilizing Fisher's exact test. Multiple genetic model analyses were applied using the Cochran-Amitage trend test to assess the association between SNPs and pCeD. All statistical tests were two-tailed, and the results were considered significant at p<0.05.

## RESULTS

### Clinical and demographic features of the groups

The clinical and demographic characteristics of the groups are presented in Table 1. The age profile of the children in the pCeD group (11.55±6.62) (mean±standard deviation) and the control group (11.76±4.92) did not show a statistically significant difference (p=0.56). When we looked at the gender distribution in the cases, the pCeD group consisted of 59.3% female and 39.7% male children, while the control group had 61% female and 39% male children, and there was no statistically significant difference between them (p=0.09).

**Table 1:** Demographic and clinical features of the groups.

Demographic and clinical features			pCeD Group (n=88)	Control group (n=84)	OR (95% CI)	p-value
Gender						
	Female		59.3%	61%	1.4 (0.72–2.73)	0.09
	Male		39.7%	39%		
	Age (M±SD)		11.55±6.62	11.76±4.92	0.37 (0.17–0.79)	0.56
Marsh classes						
	Marsh 1-2		19%	–	–	–
	Marsh 3a		22%	–	–	–
	Marsh 3b		28%	–	–	–
	Marsh 3c		31%	–	–	–
Symptoms						
	Abdominal pain		79%	–	–	–
	Inability to gain weight		58%	–	–	–
	Anemia			–	–	–
		Short height	54%	–	–	–
		Constipation	20.6%	–	–	–
		Diarrhea	15.1%	–	–	–
		Vomiting	12.9%	–	–	–
		Abdominal pain	8%	–	–	–

OR (95% CI): odds ratio (95% confidence interval).

When examining the Marsh classification of pCeD cases, it was found that 19% had Marsh 1–2, 22% had Marsh 3a, 28% had Marsh 3b, and 31% had Marsh 3c classification.

In the pCeD group, 79% of the cases had abdominal pain complaints, 58% had inadequate growth, 54% had iron-deficiency anemia, 20% had short stature, 15% had constipation, 12.9% had diarrhea, and 8% had vomiting.

In the cases classified as Marsh 3c according to the Marsh classification, pCeD cases exhibited statistical significance with respect to having iron-deficiency anemia and growth retardation (p=0.004 and p=0.03, respectively).

### Genotyping analyses

The potential relationships between the pCeD risk and the lncRNA polymorphisms (*LINC01934-rs1018326*, *IL18RAP-rs917997*, *AP002954.4-rs10892258*, *UQCRC2P1-rs6441961*, and *HCG14-rs3135316*) were investigated by comparing the genotype and allele frequency distributions of the listed polymorphisms between the groups. Genotype distributions of the polymorphisms follow the HWE. The genotype and allele frequency distributions, as well as the HWE values of the polymorphisms in the groups, are shown in Table 2.

**Table 2:** Genotype and allele frequency comparison of long non-coding RNA polymorphisms between the pCeD and control groups.

SNP and genotypes		pCeD group (n=88)	Control group (n=84)	X^2^	df	p-value
*LINC01934* **-**rs1018326						
	AA	27 (30.68%)	20 (23.81%)	9.82	2	0.007*
	AG	45 (51.14%)	32 (38.10%)			
	GG	16 (18.18%)	32 (38.10%)			
	HWE p-value	0.714	0.041*			
*IL18RAP* **-**rs917997						
	TT	11 (12.50%)	10 (11.90%)	2.42	2	0.39
	CT	40 (45.45%)	47 (55.95%)			
	CC	37 (42.05%)	27 (32.14%)			
	HWE p-value	0.970	0.126			
*AP002954.4* **-**rs10892258						
	GG	63 (71.59%)	43 (51.19%)	8.88	2	0.01*
	GA	20 (22.73%)	34 (40.48%)			
	AA	5 (5.68%)	7 (8.33%)			
	HWE p-value	0.065	0.611			
*UQCRC2P1* **-**rs6441961						
	TT	11 (12.50%)	14 (16.67%)	1.53	2	0.47
	CT	42 (47.73%)	43 (51.19%)			
	CC	35 (39.77%)	27 (32.14%)			
	HWE p-value	0.769	0.653			
*HCG14* **-**rs3135316						
	GG	78 (88.64%)	69 (82.14%)	1.98	2	0.37
	AG	7 (7.95%)	9 (10.71%)			
	AA	3 (3.41%)	6 (7.14%)			
	HWE p-value	0.000*	0.000*			
Allele frequencies		pCeD group (n=88)	Control group (n=84)	X^2^	df	p-value
*LINC01934*-rs1018326						
	T	56.25%	42.85%	3.59	1	0.05*
	C	43.75%	57.15%			
*lIL18RAP*-rs917997						
	T	35.23%	39.88%	0.46	1	0.50
	C	64.77%	60.12%			
*AP002954.4*-rs10892258						
	G	82.95%	71.43%	3.77	1	0.05*
	A	17.05%	28.57%			
*UQCRC2P1*-rs6441961						
	T	36.36%	42.26%	0.73	1	0.39
	C	63.64%	57.74%			
*HCG14*-rs3135316						
	G	92.62%	87.50%	1.46	1	0.23
	A	7.38%	12.50%			

* Significant p<0.05. HWE: Hardy-Weinberg equilibrium.

The genotypes and allele frequency distributions of the *LINC01934-rs1018326* polymorphism were statistically significant between the groups (p=0.007 and p=0.05, respectively). The AA, AG, and GG genotype distributions in the pCeD and control groups were as follows: 30.68, 51.14, and 18.18% in pCeD and 23.81, 38.10, and 38.10% in the control group, respectively. Additionally, the frequencies of A and G alleles were determined as 56.25 and 43.75% in pCeD and 42.85 and 57.15% in the control group, respectively.

The genotype and allele frequency distributions of the *IL18RAP-rs917997* variant were not found to be statistically significant between the two groups (p=0.39 and p=0.50, respectively). Distributions of TT, TC, and CC genotypes in the pCeD group were 12.50, 45.45, and 42.05%, respectively, while in the control group, they were 11.90, 55.95, and 32.14%, respectively. The frequencies of the T and C alleles were determined as 35.23 and 64.77% in pCeD and 39.88 and 60.12% in the control group, respectively.

The distributions of genotype and allele frequencies of the *AP002954.4-rs10892258* polymorphism were found to be statistically significant between the groups (p=0.01 and p=0.05, respectively). In the pCeD group, the GG, GA, and AA genotype distributions were 71.59, 22.73, and 5.68%, respectively, while in the control group, they were 51.19, 40.48, and 8.33%, respectively. The G and A allele frequency distribution in the patient group was 82.95 and 17.05%, while in the control group, it was 71.43 and 28.57%, respectively.

The genotype and allele frequency distributions of the *UQCRC2P1-rs6441961* polymorphism were not found to be statistically significant between the groups (p=0.47 and p=0.39, respectively). In the pCeD and control groups, the TT, TC, and CC genotype distributions were as follows: 12.50, 47.73, and 39.77% in pCeD and 16.67, 51.19, and 32.14% in the control group, respectively. Also, the frequencies of T and C alleles were determined as 36.36 and 63.64% in pCeD and 42.26 and 57.74% in the control group, respectively.

The genotype and allele frequency distributions of the *HCG14-rs3135316* variant were not found to be statistically significant between the two groups (p=0.37 and p=0.23, respectively). In the pCeD group, the GG, GA, and AA genotype distributions were 88.64, 7.95, and 3.41%, respectively, while in the control group, they were 82.14, 10.71, and 7.14%, respectively. Additionally, the G and A allele frequencies were determined as 92.62 and 7.38% in pCeD and 87.50 and 12.50% in the control group, respectively.

### Genetic model analyses

The *LINC01934-rs1018326* and *AP002954.4-rs10892258* polymorphisms were evaluated further by genotyping test models that include “dominant,” “recessive,” and “additive” to investigate the association of genotype and phenotype of the genes and the risk of pCeD. The *LINC01934-rs1018326* polymorphism is observed to create a 1.14-fold risk (OR: 1.14, 95%CI 0.60–2.17, p=0.004) for pCeD in the recessive model and a 1.2-fold risk (OR: 1.2, 95%CI 0.54–2.62, p=0.018) in the additive model. Similarly, the *AP002954.4-rs10892258* polymorphism is found to create a 1.40-fold risk (OR: 1.40, 95%CI 0.72–2.73, p=0.005) for pCeD in the dominant model and a 1.7-fold risk (OR: 1.7, 95%CI 1.08–2.77, p=0.015) in the additive model (Table 3).

**Table 3:** The hereditary model risk of pCeD in different genotypes of the long non-coding RNA polymorphisms.

*lncRNA SNPs*	Inheritance model	Genotype	OR (95%CI)	p-value
*LINC01934-*rs1018326	Recessive	AA+AG	Ref.	0.004*
		GG	1.14 (0.60–2.17)	
	Dominant	AA	Ref.	0.312
		GG +AG	0.65 (0.85–3.46)	
	Additive	AA	Ref.	0.018*
		AG	1.04 (0.50–2.17)	
		GG	1.2 (0.54–2.62)	
*AP002954.4-rs10892258*	Recessive	GG+GA	Ref.	0.495
		AA	0.49 (0.15–1.64)	
	Dominant	GG	Ref.	0.005*
		AA+GA	1.40 (0.72–2.73)	
	Additive	GG	Ref.	0.015*
		GA	1.03 (0.55–1.92)	
		AA	1.7 (1.08–2.77)	

* Significant p<0.05. OR (95%CI): odds ratio (95% confidence interval).

## DISCUSSION

Literature studies have shown that lncRNAs act as key regulators in inflammatory pathways. Furthermore, studies have been conducted on lncRNAs in CeD, which is also triggered by autoimmune mechanisms. The heterodimeric IL-18R formed by *IL-18R1* and the *IL-18* receptor accessory protein (*IL-18RAP*) is structurally expressed in the innate immune system cells. On the contrary, the *IL-18R1* is expressed in T cells. The *IL-18RAP* is necessary in the signaling process and its expression is increased during activation, especially in *IL-12* presence. Recently, GWAS have revealed that the *IL18RAP-rs917997* is protective in type 1 diabetes but confers susceptibility to CeD^12^. In our study, unlike the literature, no statistical association was observed between pCeD and the *IL18RAP-rs917997* polymorphism. This is believed to be attributed to the limited sample size, the possibility that different SNPs within *IL18RAP* could be responsible for CeD autoimmunity, and population variability.

The preliminary data in this study draw attention to the *LINC01934-rs1018326* polymorphism in CeD. *rs1018326* has been described as a localized SNP in the non-MHC susceptibility locus identified in ankylosing spondylitis. Plaza-Izurieta et al. and Trynka et al. in their studies found an association between *rs1018326* and CeD risk^8,11^. This study also reports a similar relationship between pCeD and the *LINC01934-rs1018326* polymorphism, suggesting that this polymorphism may be a risk factor for pCeD. Therefore, this polymorphism may have a role in inflammation in autoimmune diseases like CeD.

Ricaño-Ponce et al. identified genes near autoimmune-associated SNPs, and these SNPs were found to be particularly associated with two lncRNAs (*AP002954.4* and *AC104820.2*)^14^. In our study, similar to the literature, a statistically significant difference in genotype distribution was observed between pCeD and the *AP002954.4-rs10892258* polymorphism. Genetic model analyses revealed that this polymorphism confers a 1.40 and 1.70 times increased risk in pCeD patients.

To identify risk variants contributing to CeD susceptibility outside of the HLA-DQ region, Hunt et al. and Heel et al. determined the genotypes of the most strongly associated non-HLA markers identified in studies involving 1.643 CeD cases and 3.406 controls. The *rs6441961* polymorphism has been determined to be associated with a broad cluster of chemokine receptor genes, including *CCR1*, *CCR2*, *CCRL2*, and *CCR3*, located on chromosome 3p21^15,16^. In a study conducted by Dema et al. involving 722 Spanish CeD patients and ethnically matched 794 controls, they confirmed the association of the “A” risk allele of *rs6441961* ^17^. However, in an Italian cohort comprising 538 CeD patients and 593 healthy controls, Romanos et al. did not find any association with the *rs6441961* SNP, as previously reported by Hunt et al.^15,18^ Additionally, a meta-analysis study conducted in 2015 provides strong predictions that *IL18RAP-rs917997*, *CCR3*, or *UQCR2P1-rs6441961* may be potential risk factors for CeD in European populations^12^. In our study, similar to the results of Romanos et al., no association was found between the *rs6441961* polymorphism and pCeD. This alignment has been attributed to the fact that the patient population selected in this study is from the same European cohort. It has been suggested that discrepancies in other studies may arise from population differences across Europe in terms of loci contributing to CeD.

Santin et al. conducted a study in which they performed high-resolution SNP genotyping in the MHC region. They compared the CeD subjects with homozygous *HLA-DR3* with healthy heterozygous controls that carry one copy of preserved and extended *B8-DR3-DQ2*. Their study identified two linked SNPs. One of them was present in the *TRIM27* gene, and the other one is *rs3135366* located in the non-coding *HCG14* gene. Through the stratification studies, the *HCG14* gene demonstrated a significant correlation, which is independent of the *HLA-DR-DQ* loci. In the analysis of duodenal biopsies of the CeD patients, the epithelial *HCG14* expression was slightly downregulated. The potential associations between the downregulated expression of *NOD1* in duodenum and the polymorphisms in the *HCG14* region were suggested by the eQTL analysis^19^. In our study, unlike Santin et al., no association was detected between the *HCG14-rs3135366* polymorphism and pCeD in the analysis we conducted on duodenal biopsy samples. This situation may once again be attributed to population differences and inadequate sample size.

## CONCLUSION

The results of this study highlight the significance of the *LINC01934-rs1018326* and *AP002954.4-rs10892258* polymorphisms in pCeD and suggest that these polymorphisms might be linked to inflammation in autoimmune diseases such as CeD.
